# The Impact of Rayleigh Scattering in UWFBG Array-Based Φ-OTDR and Its Suppression Method

**DOI:** 10.3390/s23115063

**Published:** 2023-05-25

**Authors:** Feng Wang, Yong Yu, Rui Hong, Ruifeng Tian, Yixin Zhang, Xuping Zhang

**Affiliations:** 1 Key Laboratory of Intelligent Optical Sensing and Manipulation, Ministry of Education, College of Engineering and Applied Sciences, Nanjing University, Nanjing 210093, China; mg20340013@163.com (Y.Y.); dg21340004@smail.nju.edu.cn (R.H.); 522022340014@smail.nju.edu.cn (R.T.); zyixin@nju.edu.cn (Y.Z.); xpzhang@nju.edu.cn (X.Z.); 2 Shenzhen Research Institute of Nanjing University, Shenzhen 518000, China

**Keywords:** ultra-weak fiber Bragg grating (UWFBG), Φ-OTDR, distributed acoustic sensing system (DAS), Rayleigh backscattering (RBS), demodulation accuracy

## Abstract

Ultra-weak fiber Bragg grating (UWFBG) array-based phase-sensitive optical time-domain reflectometry (Φ-OTDR) utilizes the interference interaction between the reference light and the reflected light from the broadband gratings for sensing. It significantly improves the performance of the distributed acoustic sensing system (DAS) because the intensity of the reflected signal is much higher than that of the Rayleigh backscattering. This paper shows that Rayleigh backscattering (RBS) has become one of the primary noise sources in the UWFBG array-based Φ-OTDR system. We reveal the impact of the Rayleigh backscattering signal on the intensity of the reflective signal and the precision of the demodulated signal, and we suggest reducing the pulse duration to improve the demodulation accuracy. Experimental results demonstrate that using light with a 100 ns pulse duration can improve the measurement precision by three times compared with the use of a 300 ns pulse duration.

## 1. Introduction

Phase-sensitive optical time-domain reflectometry (Φ-OTDR) has garnered increasing interest in recent years as a powerful detection method. It boasts several advantages, such as quantitative measurement [1,2], a broadband response [3,4], and high sensitivity [5,6], making it widely used in geological exploration [7,8], structural health monitoring [9,10], and intrusion detection [11,12]. Despite its strengths, Φ-OTDR systems that rely on Rayleigh backscattering for sensing face limitations due to the weak intensity of RBS light. However, recent advances in the manufacturing techniques of ultra-weak fiber Bragg grating (UWFBG) arrays have allowed for significant improvements in Φ-OTDR performance [13,14]. For instance, in 2017, Fan Ai et al. achieved a broadband response of 2 Hz to 5 kHz in a section of UWFBG array with a spatial resolution of 4 m [15]. In 2018, Yi Li et al. demonstrated position-resolved low-frequency vibration sensing based on Fizeau interference in a fiber-optic system consisting of 500 identical UWFBGs with a frequency range of 0.5 Hz to 100 Hz [16]. In 2020, Chengli Li et al. proposed a hybrid UWFBG array that could measure temperature and vibration simultaneously [17]. In 2022, Yichang Wu et al. introduced a temporal differential method for large-strain measurement and effectively suppressed low-frequency deviation noise via the weighted-gauge approach [18]. Additionally, in the same year, Minghong Yang et al. proposed a large-capacity, long-distance distributed acoustic sensing system using a 54.14-km-long UWFBG array with a spatial resolution of 5 m, without the need for inline optical amplifiers. This system successfully reconstructed dynamic acoustic signals at the far end of the UWFBG array [19].

Different from the traditional Φ-OTDR systems, the carrier of vibration information in the UWFBG array is reflected light, which significantly enhances the SNR of the sensing signal. However, different types of noise still limit the system’s performance. Researchers have proposed various approaches to suppress the effects of these noise. White noise, as one of the main noise types within the UWFBG array, significantly reduces the SNR of the beat signal with increasing distance, limiting long-range sensing. Guanhua Liang et al. proposed a demodulation method for quasi-distributed acoustic sensing systems based on dual-identical-chirped-pulse and weak fiber Bragg gratings, which can reconstruct the dynamic strain well even if the SNR is reduced to 3.234 dB at the end of a 101.64-km-long fiber [20]. Meanwhile, Mengshi Wu et al. identified that a nonideal laser in UWFBG array-based Φ-OTDR would introduce phase noise to reduce the sensitivity of low-frequency signal measurements [21]. They further proposed a phase noise compensation system capable of detecting signals in the range of 10 Hz to 2500 Hz, with sensitivity of 3.84 pε/√(Hz). Polarization fading noise is another issue that can seriously diminish the visibility of the beat signal within the UWFBG array. Xuelei Fu et al. proposed a technique based on matched interference between polarization-switched pulses, which effectively suppresses the effect of polarization fading [22].

During practical experiments, we observed that RBS can have a significant impact on the reflected signal, acting as noise that impairs the system’s sensing performance. Therefore, to enhance the sensing performance of the system, it is necessary to study the influence of RBS on the reflected light in the UWFBG array-based Φ-OTDR system. In traditional Φ-OTDR systems, the quantitative measurement of disturbances is achieved by extracting the phase difference of the RBS light between two undisturbed fiber sections on each side of the disturbed region [23]. However, Reference [24] points out that when restoring the actual phase variation induced by external disturbances, the reconstructed vibration intensity is not equal to the actual vibration intensity if the selected fiber section is located in the vibrating area. In the UWFBG array, the fiber sections that generate RBS light are likely to be located in the vibrating area. Thus, when these sections produce RBS light that interferes with the reflected light, it affects the demodulation phase accuracy, leading to potential negative effects on the sensing performance.

To improve the system’s accuracy, we systematically study the RBS light’s influence on the demodulated phase in a dual-pulse Φ-OTDR system based on the UWFBG array in this paper. Our analysis reveals that the demodulation phase is significantly affected when the optical fiber is disturbed, reducing the sensing accuracy of the disturbing signal. To address this issue, we proposed a method to optimize the detection pulse duration to reduce measurement errors and effectively improve the sensing precision for vibration signals.

## 2. Principle

**Figure 1:**
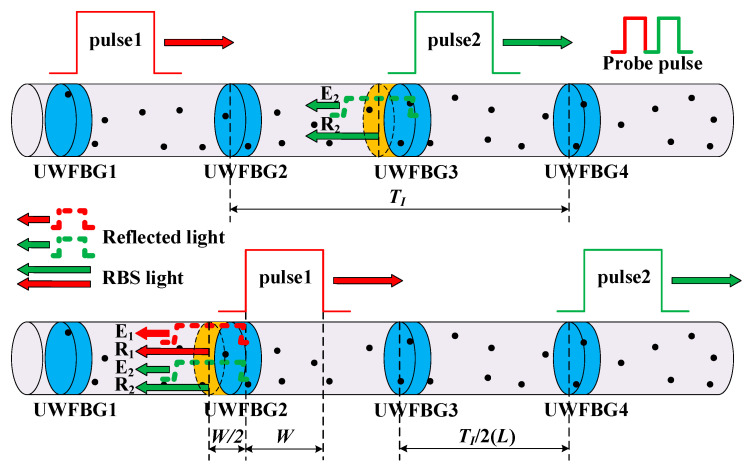
Superposition principle of reflected and RBS light in UWFBG array.

In a Φ-OTDR system based on a UWFBG array, as shown in Figure 1, we denote the spacing between adjacent UWFBGs as *L* and the reflection coefficient as *r*. The distance between UWFBG1 and the input fiber end is denoted as *z*. The frequencies of the front (pulse2) and rear (pulse1) probe pulses are denoted as *ω*_2_ and *ω*_1_. At time *t*, the reflected optical electric field generated at the adjacent weak gratings UWFBG1 and UWFBG2 can be expressed as follows [25]:

(1) $$E_{1}=E_{0}\sqrt{r}\cos(\omega_{1}t+\frac{4\pi nz}{\lambda })$$

(2) $$E_{2}=E_{0}\sqrt{r}\cos\left[\omega_{2}t+\frac{4\pi nz}{\lambda }+\varphi_{0}+\frac{4\pi n}{\lambda }(L+\Delta L)\right]$$

where *E*_0_ is the electric field of the incident pulse, *n* is the refractive index of the fiber core, *λ* is the central wavelength of the laser, and *φ*_0_ is the initial phase difference between the front and rear reflected light.

When considering the RBS, the optical fiber is regarded as a series of spatially discrete “scattering points”. Then, the RBS signals generated by these points with half the pulse width are superimposed together. Based on this model, the electric fields of the RBS light generated by the front and rear probe pulses received at time *t* can be expressed as [26]

(3) $$E_{3}=\sum_{i=1}^{N}E_{0}\exp(-\alpha \frac{c\tau_{i}}{n})a_{i}\exp\left[j\omega_{1}(t-\tau_{i})\right]\mathrm{rect}(\frac{t-\tau_{i}}{W})$$

(4) $$E_{4}=\sum_{i=1}^{N}E_{0}\exp(-\alpha \frac{c\tau_{i}}{n})a_{i}\exp\left[j\omega_{2}(t+\frac{2nL}{c}-\tau_{i})+\varphi_{0}\right]\mathrm{rect}(\frac{t+2nL/c-\tau_{i}}{W})$$

where *W* is the length of the incident light pulse in the fiber, *τ_i_* represents the time delay generated by the *i*th scattering point, *a_i_* represents the scattering coefficient of the ith scattering point, *c* is the speed of light, and *N* is the total number of the scattering points along the fiber. The superposition model between the reflected light and the RBS light in the UWFBG array is shown in Figure 1, where the particles signify scattering points in the fiber. It should be noted that the Rayleigh scattering of a probe pulse is generated by a specific fiber section with the length of half the pulse width [27]. In Figure 1, the yellow shaded area, located immediately before UWFBG3 and having a length of *W*/2, indicates the fiber section that generates the Rayleigh scattering superimposed with the reflected head of probe pulse2 at a particular time. Thus, different parts of the reflected probe pulse2 overlap with the Rayleigh scatterings generated by different fiber sections, each of which has the length of *W*/2. At time *t*_1_, the reflected light (**E_2_**) of probe pulse2 at UWFBG3 is superimposed with the RBS light (**R_2_**) and transmitted backward. At time *t*_1_ + *T_I_*/2, the probe pulse1 is reflected at UWFBG2 (**E_1_**) and superimposed with the RBS light (**R_1_**). Since the interval between probe pulse1 and probe pulse2 is *T_I_*, **E_1_** and **R_1_** also overlap with **E_2_** and **R_2_**, and the four components transmitted to the fiber’s input end together. Therefore, the resulting intensity of the optical signal is given by

(5) $$\begin{array}{ll} I(t) & =2E_{0}^{2}r\\ \; & +2rE_{0}^{2}\cos\left[\Delta \omega t+\frac{4\pi n}{\lambda }(L+\Delta L)+\varphi_{0}\right]\\ \; & +E_{0}^{2}\sum_{i=1}^{N}\exp(-2\alpha \frac{c\tau_{i}}{n})a_{i}^{2}\mathrm{rect}(\frac{t-\tau_{i}}{W})\\ \; & +E_{0}^{2}\sum_{i=1}^{N}\exp(-2\alpha \frac{c\tau_{i}}{n})a_{i}^{2}\mathrm{rect}(\frac{t+2nL/c-\tau_{i}}{W})\\ \; & +2E_{0}^{2}\sum_{j=1}^{N}\sum_{k=j+1}^{N}a_{j}a_{k}\cos\left[\omega_{1}(\tau_{j}-\tau_{k})\right]\exp\left[-\alpha \frac{c(\tau_{j}+\tau_{k})}{n}\right]\mathrm{rect}(\frac{t-\tau_{j}}{W})\mathrm{rect}(\frac{t-\tau_{k}}{W})\\ \; & +2E_{0}^{2}\sum_{j=1}^{N}\sum_{k=j+1}^{N}a_{j}a_{k}\cos\left[\omega_{2}(\tau_{j}-\tau_{k})\right]\exp\left[-\alpha \frac{c(\tau_{j}+\tau_{k})}{n}\right]\mathrm{rect}(\frac{t+2nL/c-\tau_{j}}{W})\mathrm{rect}(\frac{t+2nL/c-\tau_{k}}{W})\\ \; & +2E_{0}^{2}\sum_{i=1}^{N}\sum_{j=1}^{N}a_{i}a_{j}\cos(\Delta \omega t+\frac{2\omega_{2}nL}{c}-\omega_{2}\tau_{j}+\omega_{1}\tau_{i}+\varphi_{0})\exp\left[-\alpha \frac{c(\tau_{j}+\tau_{i})}{n}\right]\mathrm{rect}(\frac{t-\tau_{i}}{W})\mathrm{rect}(\frac{t+2nL/c-\tau_{j}}{W})\\ \; & +2\sqrt{r}E_{0}^{2}\sum_{i=1}^{N}\exp(-\alpha \frac{c\tau_{i}}{n})a_{i}\cos(\frac{4\pi nz}{\lambda }+\omega_{1}\tau_{i})\mathrm{rect}(\frac{t-\tau_{i}}{W})\\ \; & +2\sqrt{r}E_{0}^{2}\sum_{i=1}^{N}\exp(-\alpha \frac{c\tau_{i}}{n})a_{i}\cos(\Delta \omega t+\frac{2\omega_{2}nL}{c}-\omega_{2}\tau_{j}-\frac{4\pi nz}{\lambda }+\varphi_{0})\mathrm{rect}(\frac{t+2nL/c-\tau_{i}}{W})\\ \; & +2\sqrt{r}E_{0}^{2}\sum_{i=1}^{N}\exp(-\alpha \frac{c\tau_{i}}{n})a_{i}\cos\left[\Delta \omega t+\omega_{1}\tau_{i}+\frac{4\pi nz}{\lambda }+\frac{4\pi n}{\lambda }(L+\Delta L)+\varphi_{0}\right]\mathrm{rect}(\frac{t-\tau_{i}}{W})\\ \; & +2\sqrt{r}E_{0}^{2}\sum_{i=1}^{N}\exp(-\alpha \frac{c\tau_{i}}{n})a_{i}\cos(\omega_{2}\tau_{i}+\frac{4\pi nz}{\lambda }+\frac{4\pi n}{\lambda }\Delta L)\mathrm{rect}(\frac{t+2nL/c-\tau_{i}}{W}) \end{array}$$

In Equation (5), the first term corresponds to the direct current (DC) component, and the second term represents the beat signal that arises from the interference of the reflected signals, which is of interest to us. The third to seventh terms correspond to interference terms that arise from the RBS light, but we can safely ignore these terms because their intensity is low. The remaining terms correspond to the interference between the RBS light and the reflected signal. Since the reflected signal amplifies the RBS signals, we are particularly interested in the impact of these last four terms.

**Figure 2:**
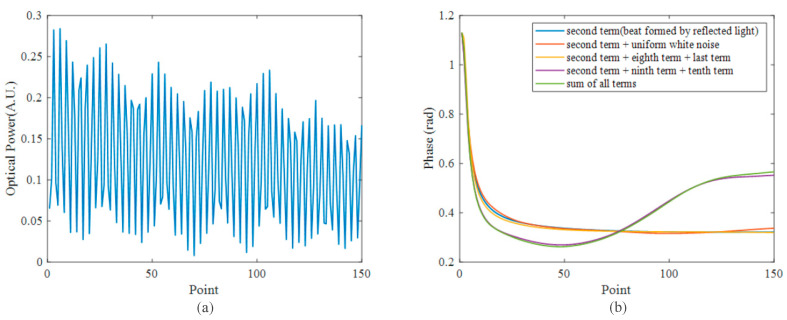
Simulation beat signal and phase. (**a**) Beat signal with uniform white noise; (**b**) phases derived from different terms.

We verified Equation (5) through simulation and compared the impact of the RBS light with that of white noise. Thus, we added white noise to the simulation results based on the SNR of the actual beat signal. The simulation parameters are summarized in Table 1. The beat signal obtained by summing all the terms in Equation (5) is shown in Figure 2a, and the phases demodulated from different terms are shown in Figure 2b. The first curve in Figure 2b corresponds to the phase obtained from the interference of the reflected signals, which remains constant for different positions. The second and third curves were obtained from the reflected signals after adding white noise or the superposition of the RBS light and the reflected light. It can be observed that the phase does not change significantly and essentially coincides with the first curve. The fourth curve corresponds to demodulation after adding the interference term formed by the RBS light and reflected light, and the fifth curve corresponds to demodulation after adding all the terms. The phases of these two curves change significantly at different positions, but they essentially coincide with each other.

Apparently, the interference of the reflected light and the RBS light causes obvious deviation in the phase signal, which may significantly impact the demodulation results. For simplicity, we call the beat signal generated by the front and the rear reflected lights the reflection–reflection beat, and we call the beat signal generated by the interference of the reflected light and the RBS light the reflection–scattering beat. Therefore, Equation (5) can be simplified as the combination of the reflection–reflection beat and the reflection–scattering beat:

(6) $$\begin{array}{l} A\cos(\omega t+\alpha )+B\cos(\omega t+\beta (z))\\ =\sqrt{A^{2}+B^{2}+2AB\cos\left[\alpha -\beta (z)\right]}\times \cos\left\{\omega t+\tan^{-1}\left[\frac{A\sin(\alpha )+B\sin(\beta (z))}{A\cos(\alpha )+B\cos(\beta (z))}\right]\right\} \end{array}$$

where *A* is the intensity of the reflection–reflection beat, *B* is the intensity of the reflection–scattering beat, *ω* is the beat frequency, *α* is the phase of the reflection–reflection beat, and *β*(*z*) is the phase of the reflection–scattering beat, which will change with the position of the RBS signal. Obviously, the combined phase is jointly determined by *A*, *B*, *α*, and *β*(*z*). Only when *A* is much larger than *B*, the combined phase can be considered as the phase of the reflection–reflection beat. When vibration occurs, it will induce additional phase changes in *α* and *β*(*z*). Thus, the final combined phase can be expressed as

(7) $$\varphi =\tan^{-1}\left\{\frac{A\sin(\varphi_{1}+\Delta \varphi )+B\sin\left[\varphi_{1}+\varphi_{2}(z)+\delta \varphi (z)\right]}{A\cos(\varphi_{1}+\Delta \varphi )+B\cos\left[\varphi_{1}+\varphi_{2}(z)+\delta \varphi (z)\right]}\right\}$$

where *φ*_1_ is the initial phase, which is affected by the laser frequency drift [29] and the inconsistent phase between the modulation signal and the trigger signal [30]; Δ*φ* is the phase variation generated by the disturbance between the two UWFBGs, which is proportional to the magnitude of the disturbance. The component *φ*_2_(*z*) is an equivalent phase, determined by the state of the massive Rayleigh scattering points. Over a certain period, the state of the Raleigh scattering point remains relatively constant. Thus, *φ*_2_(*z*) at the same position in multiple time-domain curves remains unchanged. *δφ*(*z*) describes the vibration-induced variation in the equivalent phase of the RBS light, which changes with the position in the fiber. For the scattering section represented by the yellow shaded area in Figure 1, *δφ*(*z*) equals Δ*φ*.

In the UWFBG array system, the reflection–reflection beat’s light intensity is dominant compared with the reflection–scattering beat’s light intensity. Combined with Equation (7), we can see that the main component in the synthetic phase is *φ*_1_ + Δ*φ*, and the minor component in the synthetic phase is *φ*_1_ + *φ*_2_(*z*) + *δφ*(*z*). After demodulation, the influence of the initial phase *φ*_1_ and the temporary stable phase *φ*_2_(*z*) can be removed with the differential method [1], leaving only two terms whose proportion is determined by *A* and *B*. Thus, the demodulated vibration information can be considered as the sum of the original vibration and a small vibration with the same frequency and a different phase. Since the magnitude of *δφ*(*z*) changes at different positions in the beat signal, the demodulated vibration’s amplitudes at different points in the beat signal are different. Additionally, the proportion of the reflection–scattering beat’s light intensity in the whole beat signal fluctuates over a long period, resulting in a noticeable fluctuation in the demodulated vibration amplitude during different periods.

Based on the above analyses, we propose reducing the pulse duration of the probe pulse to improve the precision in reconstructing vibrations. The intensity of the reflection–scattering beat is influenced by several factors, including the incident light intensity, the reflectivity of the UWFBG, attenuation, the polarization state, and the number of superimposed scattering points. In a fixed system, a change in the first factor will result in synchronous changes in the reflection–reflection beat and reflection–scattering beat’s intensities, while the next two terms cannot be altered. Since the fiber’s birefringence is random along its length, the polarization state of the reflected light and RBS light cannot be controlled. Therefore, we can only adjust the number of scattering points by varying the pulse duration, which alters the intensity of the reflection–scattering beat. By reducing the proportion of the reflection–scattering beat’s intensity in the overall beat intensity, we can minimize the impact of Rayleigh scattering on the demodulation phase.

## 3. Results

### 3.1. Experimental Setup

**Figure 3:**
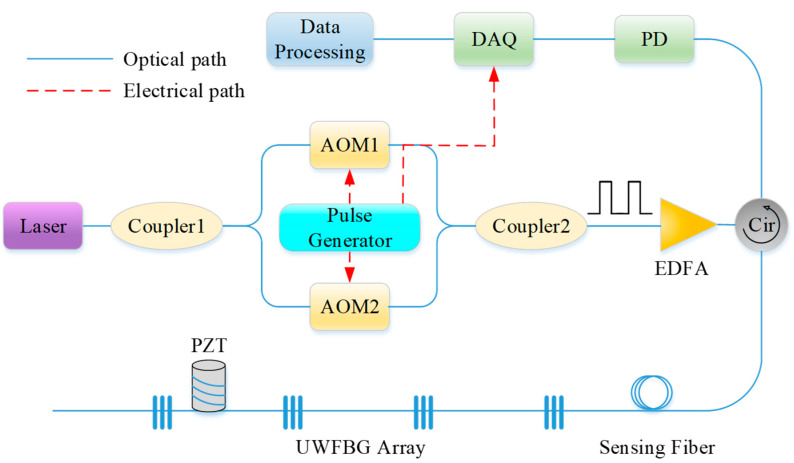
The experimental setup of the Φ-OTDR system based on UWFBG array.

The experimental setup of the proposed system is shown in Figure 3. The light source was a highly coherent laser with a linewidth of 1 kHz and a central wavelength of 1550 nm. The output CW light was divided into two branches using a 50:50 optical coupler (OC1). Then, they were modulated into optical pulses by two acousto-optic modulators (AOMs), with an extinction ratio (ER) of more than 50 dB. One AOM had a frequency shift of 40 MHz, and the other had a frequency shift of 200 MHz. Their periods were both 25 μs, and they had a time delay controlled by the pulse generator. After this, the two probe pulses were mixed in OC2 and amplified by a pulsed erbium-doped fiber amplifier (EDFA) with a gain of 23 dB. The EDFA incorporated an internal filter that could tailor its amplified spontaneous emission noise outside the 1550 ± 0.25 nm bandwidth wavelength. Then, the pulse pair was launched into a 1.5-km-long UWFBG array through an optical circulator (CIR). The distance between two adjacent UWFBGs in the fiber was 50 m. The reflectivity of the UWFBG was about −40 dB. The returned beat signal was then received by the photodetector (PD), possessing a bandwidth of 300 MHz and a response rate of 0.9 A/W to 1550 nm light waves. A data acquisition card with a sampling rate of 500 MSa/s received the electrical signals and sent them to the computer for signal processing.

### 3.2. Experimental Results and Discussion

**Figure 4:**
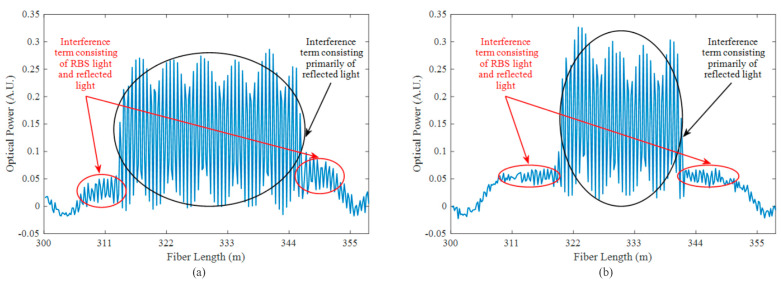
Beat signal with different pulse durations. (**a**) Beat signal formed by the interference of 450 ns and 300 ns duration pulses; (**b**) beat signal formed by the interference of 450 ns and 200 ns duration pulses.

In our experiment, we initially set the pulse durations of the front and rear pulses to 450 ns and 300 ns, respectively. Subsequently, meticulous fine-tuning of the time delay to 575 ns was implemented, carefully aligning the central positions of the two reflected lights. This configuration ensured that the edges of the long pulse’s reflected light only overlapped with the RBS light from the short pulse. The red circles in Figure 4a indicate the reflection–scattering beat signal, which has considerable intensity. The black circle in Figure 4a represents the beat signal that we need, primarily formed by the interference of the reflected light from the front and rear pulses. However, it also contains the superimposed component of the RBS light and reflected light. To demonstrate the impact of RBS, we reduced the duration of the rear pulse to 200 ns and concurrently adjusted the time delay to 625 ns, while keeping the front pulse’s duration constant. The new interference results are presented in Figure 4b. Comparing the reflection–scattering beat marked by the red circle in Figure 4a,b, we observe an increase in the intensity of the reflection–scattering beat signal as the pulse duration increases, indicating the influence of RBS.

**Figure 5:**
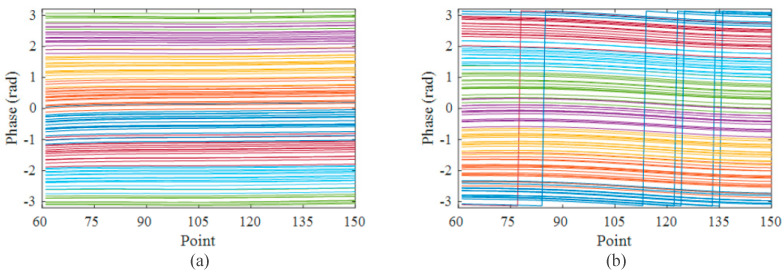
(**a**) One hundred phase curves of the first group; (**b**) one hundred phase curves of the second group 5 s after the first group.

In order to further investigate the behavior of the Rayleigh scattering points, we conducted an experiment without any external interference. Specifically, we configured both pulses to have a duration of 300 ns, set the time delay to 500 ns, and maintained a period of 25 μs. We collected two groups of 100 successive time-domain curves with a time interval of 5 s. Subsequently, we performed IQ demodulation on the acquired time-domain signals, extracting the phase information [31]. The demodulated phases for each group are presented in Figure 5a,b, respectively. Each line in the figure, distinguished by different colors, represents the phase information obtained from different time domain signals. Interestingly, we observed a high degree of similarity between the phase curves in each group, indicating that the influence of the Rayleigh scattering points on the demodulation results tended to be consistent over a short period. However, when we compared Figure 5a,b, which represent phase curves obtained from the same position, we noticed significant differences between them. These differences can be attributed to the changing state of the Rayleigh scattering points, which in turn affected the superimposed RBS light and the resulting demodulation outcome.

**Figure 6:**
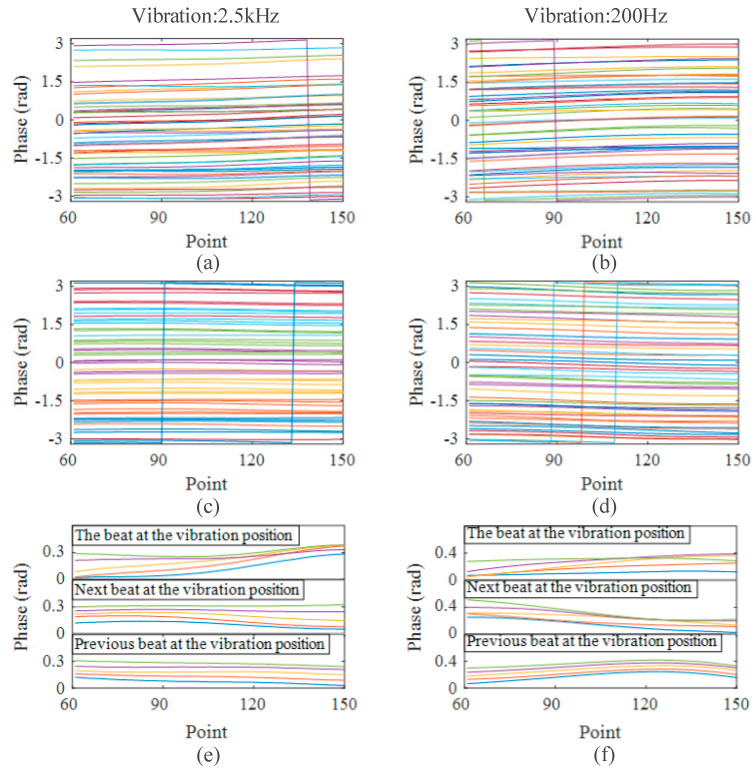
Phase curves affected by vibration: (**a**) 50 phase curves at 2.5 kHz vibration position; (**b**) 50 phase curves at 200 Hz vibration position; (**c**) 50 phase curves at 2.5 kHz vibration position; (**d**) 50 phase curves at 200 Hz vibration position; (**e**) details of the phase curves around 2.5 kHz vibration position; (**f**) details of the phase curves around 200 Hz vibration position.

Next, we applied a 10 V, 2.5 kHz sinusoidal signal on the fiber with a PZT at 500 m. The PZT was placed near the rear UWFBG between two UWFBGs. By collecting 50 successive time-domain curves and demodulating their phases, we obtained the phase curves of the beat signal between two UWFBGs at and after the vibration location. Figure 6a shows the phase at the vibrating position, and Figure 6c shows the phase obtained from the UWFBG beat signal after the vibration. The beat signal was chosen to be as close to the vibrating position as possible. As shown in Figure 6e, we vertically translated the phase curve to near 0 rad to compare the similarity of five consecutive phase curves obtained from different beat signals near the vibration location. The similarity of the phase curves before the vibration location was high. However, the similarity decreased significantly for the phase curves at the vibration location and after this. The reason is that the phase relationship between the RBS light and the reflected light before the vibration position remained unchanged, whereas the relationships at the vibration position and after this had been changed by the vibration. The greater the change, the more strongly the composite phase fluctuated.

**Figure 7:**
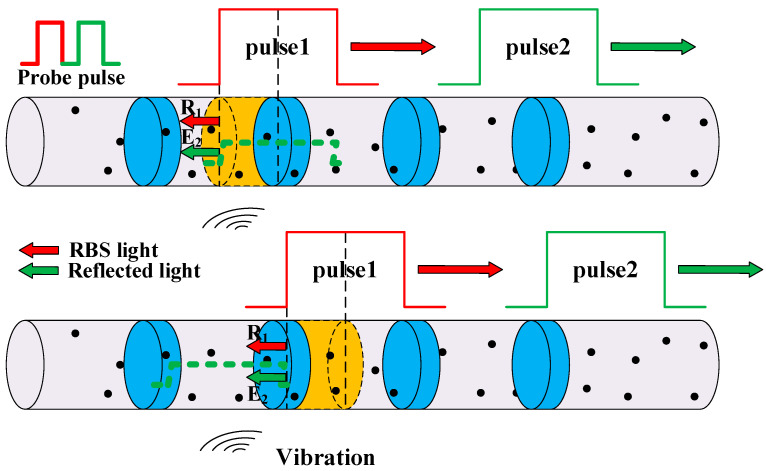
Superposition principle of reflected and RBS light in UWFBG array adjacent to vibration position.

For instance, the phase difference between the RBS light and the reflected light after the vibration position experienced a gradual decrease until it reached 0. As shown in Figure 7, during the forward propagation of the probe pulse, the scattering points covered by the rear pulse light were constantly updated. They experienced a transition from partially crossing the vibration to completely crossing it, whereas the reflected light was always generated by the reflection of the UWFBG after the vibration. As a result, the difference between the phase changes of the RBS light of the rear pulse and the reflected light of the front pulse, induced by the vibration, gradually decreased until it reached zero.

We also applied a 200 Hz sinusoidal signal to the PZT, collected the time-domain curves, and obtained the phases every 1.25 ms. Figure 6b,d show the phases demodulated from the beat signals at and after the vibration position, respectively. Similar to the previous experiment, the similarity between the phase curves at the vibration position decreased, as well as that after the vibration position. However, the similarity of the phase curves before the vibration position remained unchanged.

**Figure 8:**
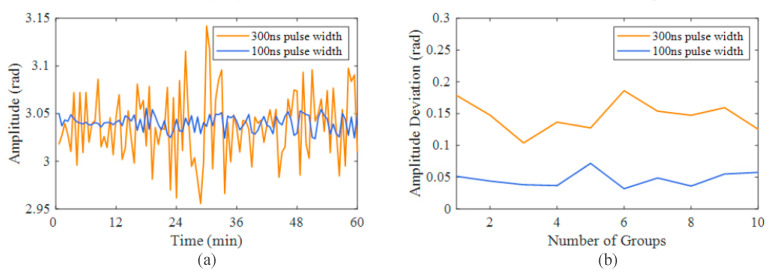
Amplitude and deviation of recovered vibration with different pulse durations. (**a**) Change in amplitude of the recovered vibration within 1 h. (**b**) Maximum amplitude deviation measured over multiple time periods.

Then, we conducted an experiment to compare the performance of two different pulse durations. A 2 V, 200 Hz sinusoidal signal was applied to the PZT, and we collected 100 groups of signals at a pulse duration of 300 ns, with an interval of 45 s. Each group consisted of 1000 consecutively collected time-domain signals. The orange curve in Figure 8a shows the amplitude change of the retrieved vibration within 1 h. It had a maximum deviation of 0.19 rad. This fluctuation was due to the variation in the proportion of the reflection–scattering beat light in the entire beat light. Next, we adjusted the pulse duration to 100 ns and repeated the experiment. The blue curve in Figure 8a shows the amplitude change of the retrieved vibration within 1 h, with a maximum deviation of only 0.03 rad. We repeated the above experiments several times in different periods. As shown in Figure 8b, the fluctuation in the vibration amplitude measured with a pulse duration of 100 ns was about 0.05 rad, which was approximately one third of the fluctuation in the vibration amplitude measured with a pulse duration of 300 ns. It is evident that the smaller the pulse duration, the more accurate the reconstructed vibration amplitude will be.

In practical applications, the selection of the pulse duration also needs to consider the hardware limitations and demodulation restriction. A smaller pulse duration requires an AOM having a higher response speed, which is difficult to realize. An insufficient response speed can cause amplitude fluctuations in the beat signal, resulting in a lower signal-to-noise ratio. On the other hand, the phase demodulation process requires sufficient effective signals. In I/Q demodulation, it requires at least one cycle with a stable beat signal for correct low-pass filtering. Overall, the selection of the pulse duration should ensure that a flat beat signal with at least one complete cycle can be collected, while also minimizing the pulse duration as much as possible.

## 4. Conclusions

In this paper, we studied the impact of the RBS on the UWFBG-based Φ-OTDR system. The addition of RBS light to the reflected light caused a deviation in the signal’s phase from its original value due to the different equivalent phases of the RBS light at different positions. Under undisturbed conditions and over a time frame of milliseconds, the phase–position curves maintained a high degree of similarity, which did not affect the measurement results. However, the presence of disturbance led to inconsistent vibration information carried by different reflection–scattering beat signals, resulting in a decrease in the similarity between the phase–position curves and ultimately affecting the accuracy of the recovered vibration amplitude. Based on our experimental results, we demonstrate that shortening the pulse duration of the probe pulse can effectively improve the accuracy of the recovered vibration signal.

## Figures and Tables

**Table 1:** The parameters used in the simulation [28].

Parameter	Value	Description
Optical fiber loss	0.2	dB/km
RBS coefficient	(0, 1)	Following Rayleigh distribution
Phase of the scattering points	(−π, π)	Following uniform distribution
Beat frequency	160	MHz
Sampling rate	500	MSa/s
Interval of UWFBGs	50	m
Probe pulse width	60	m

## Data Availability

Not applicable.
